# 胸膜间皮瘤免疫治疗研究进展

**DOI:** 10.3779/j.issn.1009-3419.2022.101.17

**Published:** 2022-04-20

**Authors:** 雨佳 迟, 奕良 刘, 军 赵

**Affiliations:** 100042 北京，北京大学肿瘤医院，北京肿瘤医院，北京市肿瘤防治研究所，胸部肿瘤内一科 Department of Thoracic Medical Oncology, Peking University School of Oncology, Beijing Cancer Hospital & Institute, Beijing 100042, China

**Keywords:** 恶性胸膜间皮瘤, 免疫疗法, 免疫检查点抑制剂, 联合治疗, 生物标志物, Malignant pleural mesothelioma, Immunotherapy, Immune checkpoint inhibitors, Combination therapy, Biomarkers

## Abstract

恶性胸膜间皮瘤（malignant pleural mesothelioma, MPM）通常预后不良，患者生存期短，且长期以来缺乏有效的治疗选择。化疗对晚期患者临床转归的改善有限，患者生存期不足1年，靶向治疗也难以觅得合适靶点。近期免疫治疗研究的发展深切改变了MPM的治疗格局，其中双免疫联合治疗在各人群亚组中均较显著改善患者生存预后，延长患者生存期，因而获批用于未经治疗、不可切除MPM患者的一线治疗。有关其他免疫联合及单药方案在MPM患者一、二线治疗中的探索亦在进行之中，如何通过预测性生物标志物筛选最佳获益人群，也是研究者关注的热点。本文将对MPM流行病学、组织学特征、发病机制、治疗策略及免疫治疗在该领域的研究进展进行解读。

## Abstract (en)

Patients with malignant pleural mesothelioma (MPM) usually present with poor prognosis and short survival period, and there has been a lack of effective treatment options for a long time. Chemotherapy has limited improvement in the clinical outcome of advanced patients (the median survival is less than one year), and it is difficult to find suitable targets for targeted therapy. Recent in-depth research on immunotherapy has changed the treatment pattern of MPM. Especially, the dual immunotherapy regimen significantly improved the survival outcome of patients across subgroups and prolonged the survival time of MPM patients. Therefore, it has been approved for unresectable MPM as first-line treatment for patients. The exploration of other mono or combo immunotherapy regimens in the first and second-line settings of MPM is also underway. How to identify the best beneficial population of each regimen through predictive biomarkers is also a hot spot for researchers. This article will focus on the most up-to-date progress of MPM epidemiology, histological characteristics, pathogenesis, treatment patterns and the advances of immunotherapy in the disease.

## 1 前言

恶性间皮瘤是一种高侵袭性、预后不良的恶性肿瘤，发病率约1.5/1, 000, 000人，常见于男性^[1]^。此前因发达国家工业化程度较高，恶性间皮瘤的发生率高于发展中国家，但近年来中国等发展中国家的发病率呈持续上升趋势。我国每年约有2, 000例新发病例，年死亡约1, 600例，年增长率约为2.5%^[1]^。据研究模型^[2]^推测，至2030年我国间皮瘤发生率将升至1.9/1, 000, 000人。恶性胸膜间皮瘤（malignant pleural mesothelioma, MPM）是间皮瘤中的主要类型，约占所有间皮瘤的90%^[3]^。由于MPM发病隐匿、潜伏周期可长达数十年，患者诊断时通常已是疾病晚期，难以手术切除。因此，MPM致死率高，患者5年生存率不足10%，中位总生存（overall survival, OS）仅9个月-12个月^[4]^。此类疾病给患者及其家庭带来严重的负担和影响。

MPM的主要危险因素为石棉暴露，约80%的患者有石棉接触史。其他的致病因素还包括某些遗传基因的突变、辐射以及猿猴病毒40等^[5]^。石棉致病机制主要为：石棉纤维浸润到正常间皮细胞中后诱发巨噬细胞在该部位的聚集，并持续释放炎性细胞因子，最终导致间皮细胞恶性转化为间皮瘤细胞。间皮瘤细胞的恶性病变进一步促使免疫抑制性微环境形成，导致免疫逃逸和血管生成，从而促进肿瘤发生发展^[6]^。基于这一致病机制，通过免疫治疗调节肿瘤微环境并避免免疫逃逸成为一种潜在的治疗选择，并逐步经过临床前、多期临床研究验证最终应用于临床。本文将阐述胸膜间皮瘤的流行病学、治疗方法以及研究进展，并将着重关注免疫治疗在胸膜间皮瘤领域的最新临床应用进展。

## 2 MPM组织学分型及预后

MPM具有高度异质性^[5]^。根据组织学分型，MPM可以划分为上皮型间皮瘤和非上皮型间皮瘤（包括肉瘤样间皮瘤、双相型间皮瘤和非常罕见的结缔组织增生型间皮瘤），其中以上皮型间皮瘤最为常见，占所有组织学分型的70%-80%。不同组织学类型的间皮瘤预后有所不同，其中上皮型间皮瘤预后最好，中位OS可达13.1个月，而肉瘤样间皮瘤预后最差，中位OS仅约4个月^[5]^。由于此前的生存期相关研究均以化疗方案作为标准治疗手段，因此，不同组织学分型亚组的预后可能仅反映该亚组对化疗的敏感程度。

## 3 MPM的现有治疗方法

此前MPM预后不良，主要原因是缺乏有效的治疗选择。2003年-2020年间，培美曲塞+铂类联合方案是MPM唯一获批的一线治疗方案，但对晚期MPM患者的临床转归改善十分有限，中位OS较顺铂单药仅延长3个月^[7]^。尽管靶向治疗手段在近二十年间飞速发展，但MPM患者似乎并未能从中获益。全面基因组研究显示，MPM中的原癌基因驱动型突变十分罕见，反之，绝大多数突变均为抑癌基因的失活性突变，如*CDKN2A*/*2B*、*BAP1*、*NF2*、*TP53*等。在原癌基因驱动的多种肿瘤中，靶向原癌基因激活性突变的治疗药物已层出不穷，但可精准靶向抑癌基因失活突变的药物研发却困难重重。因此，MPM的靶向治疗进展甚微^[7]^。2015年，一项Ⅲ期随机对照研究（MAPS）显示贝伐珠单抗联合铂类双药化疗可改善患者OS [较单用化疗风险比（hazard ratio, HR）=0.77；*P*=0.016, 7]和无进展生存期（progression-free survival, PFS）（HR=0.61, *P* < 0.000, 1），因而获得美国国立综合癌症网络（National Comprehensive Cancer Network, NCCN）指南推荐，但其3级以上血管相关不良反应事件（adverse event, AE）风险过高，且未能获得美国食品药品监督管理局（Food and Drug Administration, FDA）批准^[8]^。

近期基因检测技术的发展使研究者发现间皮瘤中的基因突变数量或远高于既往发现，包括点突变、微小缺失突变和拷贝数变异等。这些大量存在的基因突变可能导致新抗原产生，并与肿瘤浸润性T淋巴细胞的克隆增殖相关。因此，越来越多的研究提示MPM可能免疫原性较强。就组织学分型细分发现，肉瘤样间皮瘤的肿瘤微环境中存在浸润性T细胞、单核细胞、成纤维细胞和内皮细胞的富集，提示该亚型间皮瘤存在免疫细胞浸润与血管富集微环境^[7]^。此类研究以及免疫治疗在其他瘤种中的快速进展推动了免疫治疗在MPM中的探索和应用。2020年10月，双免疫联合治疗（纳武利尤单抗+伊匹木单抗方案）因在晚期MPM治疗中生存获益显著，经FDA获批上市并成为MPM新的一线治疗标准方案。

晚期MPM的二线及后线治疗尚无标准治疗方案，仅纳武利尤单抗单药基于一项Ⅱ期临床研究在日本获批。多项基于程序性死亡受体1（programmed cell death-1, PD-1）/程序性死亡配体1（programmed cell death ligand 1, PD-L1）抑制剂单药或双免疫联合方案二线或后线治疗MPM的初步研究数据提示免疫带来临床获益。NCCN指南推荐培美曲塞（1类推荐）、纳武利尤单抗±伊匹木单抗、帕博利珠单抗、长春瑞滨、吉西他滨（2类推荐）用于复发/疾病进展患者的挽救治疗，但因未获批，仍为适应证外用药，且绝大多数药物均缺乏大样本随机对照研究的支持^[9]^。

## 4 免疫治疗在MPM中的研究进展

### 4.1 一线治疗

#### 4.1.1 双免疫联合治疗（纳武利尤单抗+伊匹木单抗）

双免疫联合治疗是继含铂化疗方案后唯一获批的MPM一线治疗方案，其获批的关键研究为CheckMate 743研究^[10]^。该研究是一项随机、开放性Ⅲ期研究（*n*=605），纳入未经治疗的、组织学确诊为不可切除性MPM患者，随机分组接受双免疫联合治疗组（纳武利尤单抗3 mg/kg *Q2W*+伊匹木单抗1 mg/kg *Q6W*，最多治疗2年，*n*=303），或铂类+培美曲塞化疗组[培美曲塞500 mg/m^2^+顺铂75 mg/m^2^或卡铂曲线下面积（area under the curve, AUC）5 *Q3W*，最多治疗6个周期，*n*=302]。中位随访29.7个月时，双免疫联合治疗组患者的OS较化疗组显著改善（18.1个月 *vs* 14.1个月，HR=0.74；*P*=0.002），两组患者的2年OS率分别为41%和27%^[10]^。近期公布的3年生存随访数据进一步证实了双免疫为MPM患者带来的长期生存获益。中位随访3年（35.5个月）结果显示，两组患者的3年生存率分别为23%和15%（HR=0.73, 95%CI: 0.61-0.87）^[13]^。双免疫组中位持续缓解时间（duration of response, DOR）显著长于化疗（11.6个月 *vs* 6.7个月），患者结束双免疫治疗已约1年的情况下，双免疫组的3年DOR率达28%，而化疗组该比率为0^[13]^。双免疫方案呈现出了显著的“长拖尾”效应，且这一优势随着随访时间的延长愈加凸显。

持久的治疗反应或与双免疫联合治疗的机制有关，研究提示PD-1抑制剂与CTLA-4抑制剂的联合叠加效应远大于两者单独应用时的效应总和。联合治疗可最大程度减少耗竭表型的细胞毒性CD8^+^ T细胞比例，增加活性效应T细胞的存在比例（包括活性CD8^+^和CD4^+^效应细胞）^[11]^，并可协同刺激激活的CD8^+^ T细胞和CD4^+^ T细胞浸润至肿瘤细胞[与各抑制剂单独用药相比，T细胞浸润比例升高（*P* < 0.05）]，最终持久地刺激抗肿瘤免疫效应^[12]^。

无论MPM组织学分型如何，与标准化疗相比，患者均可从双免疫治疗中获益，但非上皮型间皮瘤患者（HR=0.48）比上皮型间皮瘤患者（HR=0.85）获益程度似乎更高^[13]^。值得注意的是，非上皮型与上皮型MPM患者接受双免疫治疗的OS类似（18.1个月 *vs* 18.2个月），因此，OS获益程度的差别其实主要来自于两亚组患者对化疗的治疗反应差异：非上皮型MPM患者对化疗相对更不敏感，接受化疗的中位OS仅为8.8个月（*vs*上皮型：16.7个月），因此在该组织型亚组的患者中，双免疫治疗相较化疗的生存优势尤为显著^[13]^。

类似地，无论患者PD-L1表达水平如何均可从双免疫治疗中获益，但PD-L1≥1%的患者OS获益更为显著（HR=0.69; *vs* PD-L1 < 1%: HR=0.94）。同样应注意到PD-L1≥1%或 < 1% MPM人群接受双免疫治疗的中位OS其实并无显著差异（18.0个月 *vs* 17.3个月），生存优势的差异主要取决于两组人群对化疗的不同治疗反应。PD-L1 < 1%人群接受化疗的OS似乎优于PD-L1阳性人群（16.5个月 *vs* 13.3个月），因而缩小了与双免疫治疗组之间的生存差异^[10]^。这一结果仍需谨慎解读，因为PD-L1表达并非本研究的分层因素，因此可能造成组间偏倚，且PD-L1 < 1%人群样本量较小。因此，PD-L1是否可作为判断患者生存预后的生物标志物，仍需要进一步的探索研究。

双免疫治疗组与化疗组的中位PFS并无显著差异（6.8个月 *vs* 7.2个月，HR=0.92），但两组PFS曲线出现交叉，即研究初期化疗组PFS获益优于双免疫治疗组，但研究8个月时两曲线到达交叉点，其后双免疫治疗组的长期生存预后显著优于化疗，且优势随随访时间延长而扩大。双免疫组的3年PFS率14%，而化疗组仅为1% ^[13]^。交叉生存曲线提示化疗的疾病控制快速但并不持久，而免疫治疗组部分患者在治疗初始阶段可能无响应，但是后续一旦有响应的患者可获得长期生存，因此按照常规比例风险模型进行统计学分析时（如*Kaplan-Meier*法计算的中位生存期），双免疫治疗由于延迟的临床效应可能导致治疗获益无法得到充分体现。此外，MPM有关PFS的影像学评估可能极具挑战，因肿瘤在连续的计算机断层扫描（computed tomography, CT）评估中缺乏清晰的边界，此时难以准确评估其真实体积变化，可能造成PFS的误判^[13]^。因此，OS在MPM中可能是更客观且可信的研究终点。

在安全性方面，免疫联合组的3级-4级治疗相关不良反应（treatment-related adverse event, TRAE）发生率与化疗组类似（31% *vs* 32%）^[13]^。3例（1%）免疫联合组患者发生治疗相关死亡事件（原因分别为肺炎、脑炎和急性心力衰竭），化疗组则有1例（< 1%，因骨髓抑制）治疗相关死亡^[13]^。纳武利尤单抗+伊匹木单抗的双免疫联合方案较标准化疗显著改善MPM患者总生存，安全性可控，因此，美国FDA于2020年10月批准其用于未经治疗、不可切除的MPM的一线治疗。中国国家药品监督管理局（National Medical Products Administration, NMPA）也于2021年6月批准纳武利尤单抗+伊匹木单抗的双免疫联合方案用于未经治疗、不可切除的非上皮型MPM一线治疗。

#### 4.1.2 PD-1/PD-L1抑制剂联合化疗

一项单臂、Ⅰ期/Ⅱ期研究^[14]^评估了度伐利尤单抗+化疗（CPPD/PEM）一线治疗初治、不可切除性MPM的疗效（DREAM, *n*=54），结果显示患者6个月时的PFS率为57%，客观缓解率（objective response rate, ORR）为48%，中位缓解持续时间为5.6个月。15%的患者发生3级-4级免疫相关不良反应，包括血脂上升、胰腺炎和肾损伤等。另一项评估度伐利尤单抗+化疗一线治疗MPM的单臂Ⅱ期研究PrE0505^[15]^也得到了相似的治疗结局，患者中位PFS和OS分别为6.7个月和20.4个月，2年PFS率和OS率分别为10.9%和44.2%，ORR达56.4%。日本研究者也在开展一项纳武利尤单抗联合标准化疗在初治、不可切除性MPM患者中的Ⅱ期探索性试验（JME-001）^[16]^，结果显示，77.8%（14/18）的患者达到完全缓解（complete response, CR）或PR，疾病控制率（disease control rate, DCR）达94.4%，且联合方案安全性可控，常见不良反应为恶心、呃逆、厌食、便秘、皮疹、贫血、疲劳等。仅1例患者因≥3级不良反应中止用药，无一例患者因不良反应死亡。此外，加拿大癌症研究组正在开展帕博利珠单抗联合化疗的Ⅱ期/Ⅲ期研究（NCT 02784171）、PrECOG在评估度伐利尤单抗联合化疗的大样本数据（DREAM3R, NCT04334759）、欧洲胸部肿瘤研究组也在探索贝伐珠单抗与阿替利珠单抗的联合方案（BEAT-meso, NCT03762018）。

### 4.2 二线治疗

#### 4.2.1 单药治疗

已有多种免疫检查点抑制剂单药正在研究之中，其中纳武利尤单抗单药已在日本获批用于治疗二线及后线MPM患者。

纳武利尤单抗的获批主要基于MERIT研究的结果。MERIT研究是一项日本单臂Ⅱ期研究（*n*=34）^[17]^，评估了有既往化疗史患者接受纳武利尤单抗治疗的疗效和安全性。其主要终点ORR达29%（10/34），且与PD-L1表达水平相关：PD-L1≥1%和 < 1%人群的ORR分别为40%和8%。MERIT研究^[17]^中患者接受纳武利尤单抗的中位PFS和OS分别为6.1个月和17.3个月。76%的患者经历TRAE但安全性可控。最短随访36个月时，患者DCR为67.6%，3年PFS率和OS率分别为12.7%和23.5%^[18]^。基于该研究，纳武利尤单抗在日本获批用于不可切除性、复发性MPM的二线治疗。NivoMes^[19]^是另一项单中心、单臂Ⅱ期研究（*n*=34），评估纳武利尤单抗单药在接受至少一种化疗方案后复发的MPM患者中的疗效和安全性，研究提示47%（16/34）患者在研究12周时可达到疾病控制，其中8例为部分缓解（partial response, PR），8例为疾病稳定（stable disease, SD）。在该人群中，PD-L1表达水平不影响治疗反应。这一生物标志物探索结果与MERIT研究相悖，但由于两项研究样本量均较小，难以有所定论。

上述研究推动了Ⅲ期随机对照研究CONFIRM的开展，332例经治、不可切除、组织学确诊的恶性间皮瘤患者随机分配至纳武利尤单抗组（*n*=221, 240 mg, *Q2W*）或安慰剂组（*n*=111）^[20]^。该研究入组人群主要为3线及后线患者，免疫治疗组3线以上患者占比71%，安慰剂组比例为67%。结果显示，纳武利尤单抗组与安慰剂组的中位OS分别为9.2个月 *vs* 6.6个月（HR=0.72, *P*=0.018），研究者评估的PFS分别为3.0个月 *vs* 1.8个月（HR=0.61, *P* < 0.001）。两组的12个月OS率分别为39.5%和26.9%；12个月PFS率则分别为14.5%和4.9%。两组患者的治疗相关不良反应发生率类似，纳武利尤单抗组和安慰剂组的≥3级AE发生率分别为45%和42%，两组分别有3.6%（*n*=5）和5.3%（*n*=4）患者发生严重AE导致死亡^[20]^。

就亚组分析而言，PD-L1≥1%人群中纳武利尤单抗与安慰剂组的中位OS分别为8.0个月 *vs* 8.7个月（HR=0.95），在PD-L1 < 1%人群中则分别为9.0个月 *vs* 6.4个月（HR=0.74, *P*=0.115）^[20]^。纳武利尤单抗在PD-L1阴性人群中的获益似乎更为明显，这一结果与其他大部分免疫检查点抑制剂单药研究和双免疫联合治疗结果不一致。鉴于该分层分析的统计学效能不足，并不具有显著统计学差异（*P*均 > 0.05），且两亚组人群安慰剂组的结果差异较大（8.7个月 *vs* 6.4个月），提示可能存在人群偏倚，因此对这一结果仍需谨慎解读。组织学亚型的分析结果也与一线双免疫联合治疗有所差异，本研究中上皮型间皮瘤患者接受纳武利尤单抗较安慰剂的获益显著（中位OS：9.4个月 *vs* 6.6个月，HR=0.71，*P*=0.021），而非上皮型间皮瘤患者接受纳武利尤单抗治疗并无显著获益（中位OS：5.9个月 *vs* 6.7个月，*P*=0.572）^[20]^。可能提示对于恶性度高、进展快的非上皮型间皮瘤患者，免疫治疗在免疫功能良好的初治患者中使用的获益会优于后线患者。

一项帕博利珠单抗单药、Ib期研究KEYNOTE-028^[21]^（*n*=25）证实20%（5/25）既往经治的MPM患者经帕博利珠单抗治疗后可达PR，52%（*n*=13）可达SD，无治疗相关死亡或导致治疗中止的不良事件。另一项芝加哥协作组开展的Ⅱ期研究（NCT02399371, *n*=65）^[22]^则显示，经治MPM患者接受帕博利珠单抗治疗后，19%患者可达到PR，无预期外的不良反应。ORR与PD-L1表达似乎相关，PD-L1≥50%、1%-49%和 < 1%人群的ORR分别为31%、26%和7%。患者的中位PFS和OS分别为4.5个月和11.5个月^[22]^。此后Ⅲ期研究PROMISE-meso（*n*=144）^[23]^比较了帕博利珠单抗与研究者选择的化疗方案在经治晚期MPM患者中的疗效，但未能达到其主要终点：尽管帕博利珠单抗组患者ORR显著较高（*vs*化疗组：22% *vs* 6%，*P*=0.004），但无论是总人群或PD-L1各分层人群的PFS或OS都未能得到显著改善（总人群中位PFS：2.5个月 *vs* 3.4个月，HR=1.06，*P*=0.76；OS：10.7个月 *vs* 11.7个月，HR=1.04，*P*=0.85）。

Avelumab的Ib期单药研究（JAVELIN Solid Tumor Trial）^[24]^纳入53例经治MPM患者，结果显示总体人群的ORR为9%，PD-L1阳性和阴性人群（以5%为阈值）ORR分别为19%和7%。总体人群的中位PFS为4.1个月，中位OS为10.7个月。9%的患者发生3级-4级治疗相关不良事件，无治疗相关死亡。

Tremelimumab的Ⅱ期双盲、安慰剂对照研究（DETERMINE, *n*=571）^[25]^初步疗效分析显示其后线治疗MPM患者的ORR为4.5%，研究未能达到其主要终点，Tremelimumab较安慰剂未能显示出在OS上的显著延长或改善（7.7个月 *vs* 7.3个月，HR=0.92）。

#### 4.2.2 双免疫联合治疗

纳武利尤单抗和伊匹木单抗的双免疫联合方案依旧是联合治疗的关注重点。一项随机、非比较性Ⅱ期研究（IFCT-1501 MAPS2, *n*=125）^[26]^将经治MPM患者随机分配至双免疫联合组或单药治疗组，结果显示两组患者12周的DCR分别为50%和44%，ORR分别为28%和19%。分析显示，高PD-L1表达（≥25%）患者的治疗反应似乎更佳，ORR可达65%-71%。但该研究在PD-L1表达评估上存在一定限制，一是高表达患者样本量较小（仅23例）；二是PD-L1表达检测应用了两种不同抗体：SP-263（*n*=125）和Dako 28-8（*n*=99），检测一致性上的差异或不能完全避免。因此，有关高PD-L1表达人群的亚组结果仍需谨慎解读。在安全性方面，联合治疗的AE发生风险较单药增加，≥3级AE发生率分别为26%和14%。联合组有3例（5%）患者发生治疗相关死亡（主要原因为肝炎、脑炎和急性肾衰竭）^[26]^。

另一类似的Ⅱ期单臂研究（INITIATE）^[27]^也评估了该双免疫联合方案在至少接受过一种铂类化疗后疾病进展的难治性MPM患者中的疗效，结果显示29%（*n*=10）患者达到PR，38%（*n*=13）患者达到SD，总DCR达68%。该研究中肿瘤PD-L1表达水平也与联合治疗的疗效相关。两项研究结果的一致性再次验证了纳武利尤单抗联合伊匹木单抗用于恶性胸膜间皮瘤二线或后线治疗的疗效和安全性。纳武利尤单抗单药或联合伊匹木单抗也被指南推荐用于二线及后线患者治疗选择^[9, 28, 29]^。

此外，NIBIT-MESO-1试验（*n*=40）^[30]^评估了Tremelimumab和度伐利尤单抗双免疫联合方案在不可切除性MPM患者二线治疗中的疗效，28%（11/40）患者显示出免疫相关ORR（immune-related ORR, irORR），DCR为65%，基线肿瘤PD-L1水平与irORR不相关^[30]^。患者中位PFS和OS分别为5.7个月和16.6个月，18%的患者发生3级-4级TRAE。

## 5 免疫治疗相关生物标志物探索

某些具有一定敏感性和特异性的免疫组织化学标志物已应用于MPM的诊断，如钙网膜蛋白和WT1，但MPM的疗效预测性生物标志物尚未能明确^[3]^。PD-L1表达水平已广泛应用于PD-1/PD-L1抑制剂的疗效预测，但仍存在诸多争议（即并非在所有临床环境下对所有免疫治疗的疗效均可准确预测），低PD-L1表达患者也有可能从纳武利尤单抗或阿替利珠单抗治疗中同等获益^[31]^，且非小细胞肺癌中的数项双免疫联合研究均提示其预测效应在双免疫临床环境下有所削弱。如CheckMate 227和CheckMate 9LA都表明无论PD-L1表达如何，双免疫或者双免疫联合化疗都可比化疗组获得显著生存获益^[32]^。

MPM中有关PD-L1表达的预测效能的探索则更在起步阶段^[33]^。首先，不同MPM标本中的PD-L1表达谱迥异，PD-L1阳性率可从20%-70%不等。可能有多重原因造成这一现象：①PD-L1抗体检测的不一致性，除常用的SP-263外，其他检测抗体还包括E1L3N和28-8等；②肿瘤本质上可能是高度异质性的，采样和检测的限制可能导致样本的选择偏倚；③不同组织学亚型可能影响其表达率，如非上皮型间皮瘤特别是肉瘤样间皮瘤中，PD-L1表达水平显著较高；④不同研究中PD-L1阳性判断的阈值也有所不同；⑤目前有关PD-L1阳性率及其预测效应的数据均来自一些小样本量的小型研究，数据可信度有限，需要更多大型研究来验证其真实表达及与有效性的相关性^[33]^。

CheckMate 743的3年随访分析还进一步对其他生物标志物进行了探索，包括4基因炎症特征评分[4-gene inflammatory signature score，包括CD8A、STAT1、LAG3，以及CD274（PD-L1）基因、肺免疫预后指数（lung immune prognostic index, LIPI）、肿瘤突变负荷（tumor mutational burden, TMB）]。研究^[13]^发现高4基因炎症特征评分与双免疫组生存获益显著改善相关。将所有检测样本的中位值作为截断值，双免疫组高评分和低评分人群的中位OS分别为21.7个月和16.8个月（HR=0.57）；但在化疗组未观察到这种相关性（HR=1.14）。而LIPI和TMB与生存获益无关。

## 6 总结与展望

MPM疾病罕见且治疗选择少，近二十年来MPM的临床治疗进展甚微，患者临床需求亟待满足。基因检测技术逐渐揭示了MPM“免疫原性”的肿瘤微环境性质，并逐渐推动免疫治疗在该领域的发展，从而革新了这类罕见患者的治疗格局。2020年纳武利尤单抗联合伊匹木单抗的双免疫治疗方案获批用于MPM的一线治疗，极大程度上改变了MPM的临床治疗现状，为患者提供了更多治疗选择。不同组织学类型胸膜间皮瘤对化疗的敏感性不同，非上皮型MPM对化疗极度不敏感；但对于一线双免疫治疗，上皮型和非上皮型MPM可得到同等获益。MPM的二线治疗选择匮乏也是临床难点之一，对于前序已经接受过化疗治疗的MPM患者，目前尚无标准治疗方案，但现有的Ⅰ期/Ⅱ期临床试验数据提示免疫治疗也可成为该类患者的治疗选择。

生物标志物的探索是免疫治疗的热门话题。根据目前的数据来看，PD-L1水平在MPM的免疫治疗中更多是作为一个预后生物标志物，而不是免疫治疗疗效的理想预测型生物标志物。而且，目前对于MPM中PD-L1的表达评估也没有统一的标准。根据CheckMate 743最近公布的生物标志物探索结果提示TMB不能有效预测免疫治疗OS获益，但对PFS获益是否有提示作用尚不得而知。虽然CheckMate 743研究的探索性分析发现4个炎症相关基因的高表达可有效预测双免疫一线治疗MPM的疗效，但该标志物应用到临床尚需要更多的研究数据。目前，除双免疫联合治疗在一线的应用外，免疫联合或单药方案的二线治疗以及多种新兴靶点探索的相关研究也正在进行之中。此外，生物标志物的进一步探索和应用仍是重点，以便更好地筛选出最佳获益人群，达到个体化精准治疗的目的。多学科协作对免疫联合或单药治疗的长期安全性管理日益规范，也将减少临床医生对其在真实世界临床环境中的用药顾虑，进一步推动其临床应用。
